# A Scoping Review of Precision Medicine in Breast Reconstruction (2011–2025)

**DOI:** 10.3390/jpm15050178

**Published:** 2025-04-28

**Authors:** Zain Aryanpour, Alec S. McCranie, Jason W. Yu, Julian Winocour, Katie G. Egan, David Mathes, Christodoulos Kaoutzanis

**Affiliations:** Division of Plastic and Reconstructive Surgery, Department of Surgery, University of Colorado Anschutz Medical Campus, Aurora, CO 80045, USA; zain.aryanpour@cuanschutz.edu (Z.A.); alec.mccranie@cuanschutz.edu (A.S.M.); jason.yu@cuanschutz.edu (J.W.Y.); julian.winocour@cuanschutz.edu (J.W.); katie.egan@cuanschutz.edu (K.G.E.); david.mathes@cuanschutz.edu (D.M.)

**Keywords:** precision medicine, plastic surgery, breast reconstruction

## Abstract

**Background:** Personalization of medical care is a significant topic of interest. Precision medicine denotes customized medical treatments based on individual genetic, molecular, and/or biomarker data. We conducted a scoping review to identify studies exploring precision medicine in breast reconstruction. **Objectives:** (1) To map the existing literature, (2) to identify key concepts, and (3) to discuss current and future clinical implications of precision medicine in breast reconstruction. **Eligibility criteria:** Indexed journal articles (primary research studies) relating to precision medicine in breast reconstruction written in the English language. **Sources of evidence:** Medline (via Pubmed), Web of Science, and the Cochrane Library. **Charting methods:** Data charting of selected studies was performed independently by two reviewers using Microsoft Excel. Any discrepancies in data charting were addressed through inter-reviewer discussion and/or expert review. **Results:** Of 321 initial records, 9 studies that were published between 2011 and 2025 were included in the final review. Eight studies focused predominantly on genomics, and one study focused predominantly on targeted therapies. Genomic-based studies were frequently implemented to evaluate patient risk and inform clinical decision-making, while targeted therapies were used to optimize reconstructive outcomes through cell-based therapies. **Conclusions:** There is a limited but emerging body of literature on precision medicine in breast reconstruction. Genomic data are the driving force of precision medicine in breast reconstruction, and multiple potential avenues exist to achieve translational applications in the short-term period. Future efforts should focus on translating known genomic data into real-time clinical applications and investing in precision-based research for targeted therapies and regenerative medicine in breast reconstruction.

## 1. Introduction

Personalization of medical care is a significant topic of interest and has historical roots in pharmacogenomics (e.g., identification of cytochrome complexes and drug interactions), HLA-typing in transplantation surgery, and the identification of oncogenes and development of targeted therapies (e.g., Herceptin and trastuzumab in breast oncology) [1,2,3]. Personalized care is the fundamental basis of clinical plastic and reconstructive surgery, as all procedures (whether cosmetic or reconstructive) are custom-tailored to patient anatomy as well as functional and aesthetic goals. Personalized medicine can be defined as an approach to healthcare that moves away from a “one size fits all” approach to tailoring care based on individual patients and specifically population-based risk factors (e.g., socioeconomic status, body mass index, age, etc.) [4]. In contrast, precision medicine is a more focused, emerging field that utilizes individual-level genomic, molecular, and/or biomarker data to guide diagnosis, risk stratification, and therapeutic decision-making [5]. While plastic surgery has historically been rooted in the principles of personalized care—through the identification of clinical risk factors, innovation in flap design, and the development of novel biomaterials—the incorporation of biologic or molecular data to direct surgical approaches is still a novel concept.

The principles of breast cancer treatment also rely significantly on individualization of care, whether at the time of tumor diagnosis, chemotherapy selection, the decision for the type of oncologic resection and need for radiation, as well as the decision for any type of post-oncologic breast reconstruction. While oncologic management has embraced precision medicine, its integration into post-oncologic breast reconstruction remains limited. There are minimal data on the state of precision medicine in plastic and reconstructive surgery, with most studies focusing on personalized care and the role of artificial intelligence in the practice of plastic surgery, including virtual surgical planning, development of risk stratification algorithms, and surgical education [6,7,8]. While artificial intelligence and virtual surgical planning can be utilized as a tool in personalized and precision medicine, these modalities generally do not consider the individual patient at the genomic, molecular, or biomarker level. Personalized care has long been a goal in surgery, but integrating genomic and molecular data into tailored surgical approaches remains an emerging field with limited research. As precision medicine is a complex and emerging field in the surgical specialties, specifically in plastic and reconstructive surgery, this article aims to provide a scoping review of the literature on precision medicine in breast reconstruction. A scoping review format was chosen given the emerging nature of this topic, allowing for the inclusion of a wider range of study designs and conceptual frameworks. The objectives of this scoping review are (1) to map the existing literature, (2) to identify key concepts, and (3) to discuss current and future clinical implications of precision medicine in breast reconstruction. We hypothesize there will be a dearth of studies regarding precision medicine in breast reconstruction.

## 2. Materials and Methods

This scoping review was performed in accordance with the Preferred Reporting of Systematic Review and Meta-Analysis for Scoping Reviews (PRISMA-ScR) guidelines. The protocol for this scoping review was registered through the Open Science Framework website (https://doi.org/10.17605/OSF.IO/BTQ74). Two authors (Z.A. and A.S.M.) conducted a comprehensive search of three major bibliographic databases, including Medline (via Pubmed), Web of Science, and the Cochrane Library, initially in January 2025 and again in March 2025, to identify relevant studies regarding precision medicine and breast reconstruction. The main search strategy was developed on Medline (via Pubmed). The following query was searched on Pubmed: (“Precision medicine” OR “personalized medicine” OR “genomic medicine” OR “tailored treatment” OR “patient-specific medicine” OR “pharmacogenomic” OR “biomarker” OR “genetic profile” OR “genetic profiling” OR “gene” OR “genomic” OR “molecular profile”) AND (“breast reconstruction”). Similar searches were conducted through the Web of Science and the Cochrane Library.

Eligibility criteria included indexed journal articles (primary research studies) relating to precision medicine in breast reconstruction written in the English language. No restrictions on publication year were applied. After the removal of duplicates, screening of records was performed based on exclusion criteria. Primary exclusion criteria included studies focusing on non-reconstructive surgery, primarily oncology focus, reviews without primary data, case reports, and expert opinions. Secondary exclusion criteria included studies focused predominantly on personalized medicine rather than precision medicine as defined previously, as well as basic science studies that do not encompass the values of precision medicine. Although personalized medicine and precision medicine are interrelated, for the purposes of this review, we applied a specific distinction to ensure consistency with our study objectives. Personalized medicine refers broadly to tailoring healthcare based on individual characteristics such as demographics, lifestyle, and patient-reported preferences [4]. Alternatively, precision medicine is defined more narrowly as approaches incorporating genomic, molecular, and/or biomarker-based data to guide diagnosis, treatment, and/or reconstructive strategy [5]. Studies that focused exclusively on personalized care—such as those describing variations in technique based on population-specific risk factors (e.g., body mass index, smoking, etc.) or patient preference without molecular or genomic considerations—were excluded. Any study in which the line between personalized and precision medicine was unclear was reviewed in full and discussed among reviewers until a consensus was reached. Data charting of selected studies was performed independently by two reviewers (Z.A. and A.S.M.) using Microsoft Excel. Any discrepancies in data charting were addressed through inter-reviewer discussion. Critical appraisal of individual sources of evidence was not performed. Descriptive statistics and qualitative results were summarized in the form of tables and figures.

Variables of interest included manuscript demographics (author, publication year), study design, population, interventions, outcomes measured, key findings, precision medicine relevance, precision medicine content category, and study classification. Precision medicine study classifications include foundational studies and clinical practice studies [9]. Foundational studies were defined as those focusing on basic science, preclinical models, and/or translational research relevant to precision medicine in breast reconstruction, such as genomic profiling, development of biomaterials, and regenerative techniques. Clinical practice studies were defined as those involving human subjects and reporting on the use of precision medicine approaches in clinical decision-making, surgical planning, and/or outcomes. Classification was performed independently by two reviewers based on these predefined criteria, with discrepancies resolved through discussion and consensus with the senior authors. This framework allowed us to distinguish between theoretical or experimental contributions to the field and those with direct clinical applications. Both study classifications were included in the final analysis, as we hypothesized a dearth of clinical application studies of precision medicine in breast reconstruction. Precision medicine content categories are not well defined in the current literature. Through literature review, we developed a simplified categorization system to denote precision medicine categories as involving either predominantly (1) genomics (e.g., pharmacogenomics, transcriptomics, proteomics, biomarkers, molecular pathways, interactions at the cellular level, etc.), (2) targeted therapy (including immunotherapy), and (3) regenerative therapy (including stem-cell-based therapies) [10].

## 3. Results

**Figure 1:**
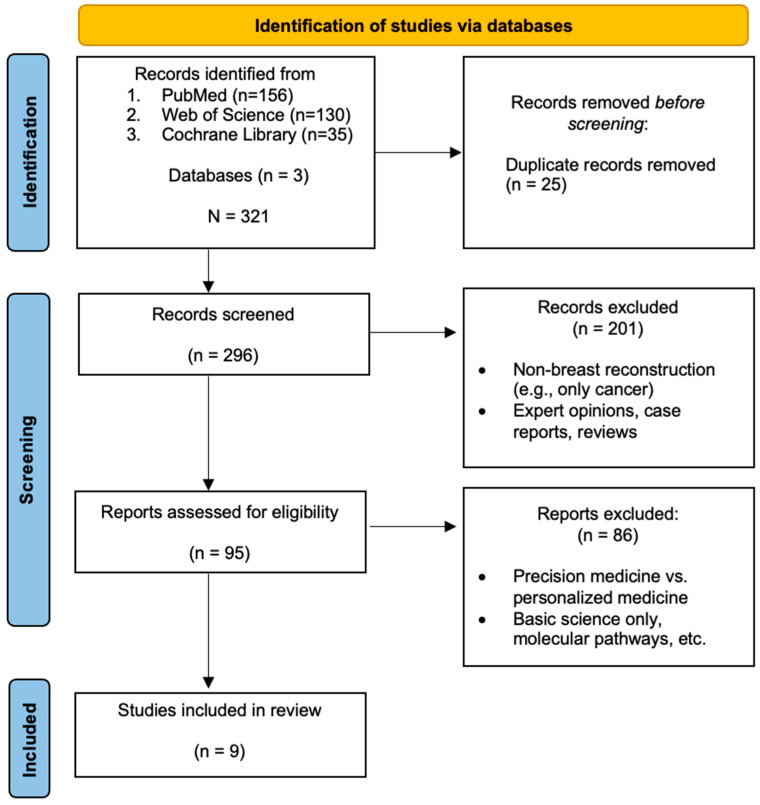
Flow chart of study identification process.

A flow chart illustrating the study article selection is available in Figure 1. Initial identification of records produced a total of 321 records; after the application of eligibility and screening criteria, nine articles were included in the final analysis. Data regarding key article characteristics, including precision medicine content category and study findings, are available in Table 1. The included articles were published between the years 2011 and 2025. Regarding the distribution of precision medicine content categories, eight studies (88.9%) focused predominantly on genomics and one study (11.1%) focused predominantly on targeted therapies; there were no studies involving regenerative therapies meeting inclusion criteria. Seven studies were primarily clinical and reported data on real patients, and two studies used animal models. Eight studies were classified as foundational in precision medicine, and one study was classified as clinical practice in precision medicine.

### 3.1. Genomics

Most studies focusing on precision medicine in breast reconstruction highlighted advances in genomics and related disciplines. Hickey et al. found that in women undergoing breast reconstruction, single nucleotide polymorphisms in the catechol-O-methyltransferase (COMT) gene were associated with persistent postsurgical pain; this highlights the potential for preoperative genetic screening to tailor pain management strategies for patients who may have this mutation [11]. Nguyen et al. found five potential biomarkers (Prol1, Muc1, Fcnb, Il1b, and Vcsa1) of flap ischemia in a rat model, highlighting the potential for real-time patient-specific flap or mastectomy flap monitoring based on these personalized biomarkers [12]. Sandberg et al. found that breast cancer subtype (e.g., luminal A, B, HER2, HER2-enriched, triple-negative, etc.) can influence the timing (i.e., immediate vs. delayed) of breast reconstruction [13]. Basta et al. found that the biomarker alpha defensin-1 can be used to detect periprosthetic breast implant infections and guide early identification and management of infections in implant-based breast reconstruction patients [14]. Frisell et al. found multiple genes associated with capsular contracture in radiated breasts, as well as those radiated breasts had increased B-cell inflammation and infiltration; as it applies to precision medicine, these findings suggest that identification of patient-specific biological factors (i.e., genes) may guide the development of tailored strategies to prevent and/or treat capsular contracture in breast reconstruction patients [16]. Mao et al. found that PRKAR2B may be a novel diagnostic biomarker for breast capsular contracture, highlighting patient biomarkers that may help predict, diagnose, and potentially prevent capsular contracture in breast reconstruction [17]. In addition, Anker et al. found that in patients undergoing autologous breast reconstruction, patients in a restrictive fluid administration group had higher TIMP-2 and IGFBP-7 biomarkers, indicating increased kidney stress; development of tailored fluid management strategies to mitigate the risk of kidney injury based on biomarkers in patients undergoing breast reconstruction highlights the utility of this study in precision medicine in breast reconstruction [18]. Miller et al. found that in patients undergoing breast reconstruction after radiation, CLCA2, COL4A5, and COL6A6 genes were associated with decreased vascularity in radiated breast capsules, that immune-related genes CXCL9 and PTCHD4 were upregulated, and several keratin-related genes were downregulated in the skin; these findings offer a foundational basis to guide tailored treatment strategies to prevent and/or treat radiation-induced fibrosis [19].

### 3.2. Targeted Therapies

Davis et al. conducted an ex vivo experimental study utilizing a rat model to create a locoregional model of breast cancer recurrence [15]. They proceeded with reconstruction using autologous tissue modified with a viral vector containing IFN-γ (experimental group) as well as a sham reconstruction group (control group). They found significantly decreased tumor burden and increased survival in the experimental group. In the context of precision medicine in breast reconstruction, these findings suggest that autologous tissue can be augmented with targeted immunotherapy to reduce breast cancer recurrence in candidate patients, especially in patients with certain breast cancer types.

## 4. Discussion

This is the first review of precision medicine in breast reconstruction. There is a limited but emerging body of literature on this topic, and most existing studies serve as foundational rather than clinical practice studies of precision medicine in breast reconstruction. Key concepts in precision medicine in breast reconstruction pertain to genomics, targeted therapies, and potentially regenerative therapies. Genomic-based studies were frequently implemented to evaluate patient risk and inform clinical decision-making, while targeted therapies were used to optimize reconstructive outcomes through cell-based therapies. Despite the limited existing data on this topic, our study highlights multiple areas of improvement and potential innovation and clinical application in precision medicine and breast reconstruction as a translational science.

### 4.1. Expanding Past Genomics

Most included precision medicine studies (8/9, 88.9%) focused on genomics, which aligns with broader trends in precision medicine, oncology, and surgical specialties [20,21,22]. While genomic data are often the catalyst for the development of targeted therapies, this suggests a relative under-utilization of other precision medicine domains within breast reconstruction. The heavy emphasis on genomics in breast reconstruction may reflect early adoption patterns but also highlights opportunities to broaden research to include regenerative medicine and targeted therapies that fall within the scope of precision medicine.

As noted previously, most of the identified studies in the review were foundational in precision medicine and require further development to be implemented in clinical practice. For example, genetic screening for single nucleotide polymorphisms in the COMT gene of patients undergoing breast reconstruction will allow the identification of patients at high risk for persistent post-mastectomy pain and thus allow for the development of tailored peri-operative pain regimens [11]. Biomarkers of flap ischemia may be augmented and/or optimized and applied similarly for monitoring of mastectomy flap necrosis [12]. Similarly, the development of a laboratory test to screen for variations in the PRKAR2B gene may allow for the identification of patients who are at high risk for capsular contracture, as well as targeted preventative and therapeutic strategies [17]. The one study that utilized precision medicine in the clinical practice of breast reconstruction tailored breast reconstruction decisions based on the molecular subtype of the patient’s cancer [13]. They found that certain subtypes of breast cancer had different profiles as it pertains to survival, recurrence, and need for post-mastectomy radiation and that surgeons should use the patient’s molecular cancer subtype to determine the timing of reconstruction. While decision guidance for breast reconstruction was limited to timing (immediate versus delayed), this study sets the framework for the clinical practice of precision medicine in breast reconstruction through utilization of patient (and cancer) genomics. Genomics is a significant tool in precision medicine in breast reconstruction and not only provides a source of potentially biomarker-driven applications, but also serves as the basis for targeted and regenerative therapies. This review found only a single study focused on targeted therapies within precision medicine and breast reconstruction. While breast cancer recurrence after mastectomy and reconstruction is relatively uncommon, it is associated with significant physical and psychological ramifications for patients [19]. Therapeutic breast reconstruction using targeted therapies (such as IFN-γ treated flaps) may become the cornerstone for preventing recurrence after reconstruction [15]. This would be especially true if targeted therapies were tailored to patients’ and/or their tumors’ molecular characteristics.

### 4.2. Next Steps in Regenerative Therapies

There was a significant deficiency of information regarding regenerative therapies and precision medicine in breast reconstruction. Regenerative therapies are historically the basis of many plastic and reconstructive surgery procedures, including fat grafting, tissue engineering of biological scaffolds, and wound healing [23]. Many potential studies were identified during the screening process; however, none of the studies focused on regenerative therapies met the inclusion criteria for precision medicine. The current literature on regenerative therapies in breast reconstruction focuses on a wide array of topics, mostly centering on molecular mechanisms of adipocytes and stem cells and potential clinical applications [24]. One study compared the genetic profiles of fat stem cells from the breast, abdomen, and face and found that fat stem cells from the breast and abdomen were most similar, suggesting that fat transfer from the abdomen to the breast is a favorable process [25]. Other studies evaluate the interactions between fat stem cells, normal breast cells, and breast cancer cells to evaluate oncologic safety [26,27]. Some studies focus on the effect of stem cells and their potential to prevent and treat capsular contracture [28]. Other studies highlight the utility of bioprinting scaffolds to improve the differentiation and survivability of adipose-derived mesenchymal stem cell grafts for improved breast reconstruction outcomes [29,30]. Despite multiple advances toward utilizing stem cell-based therapies in breast reconstruction, none of the evaluated studies considered tailoring treatments to the patient’s genomic, molecular, or biomarker data. There is a significant opportunity for tailoring regenerative medicine-based concepts to precision medicine applications, including patient-specific stem cell harvesting and customization of growth factors.

Despite promising data, regenerative medicine efforts lag behind genomics and targeted therapy, likely due to limited long-term outcomes data, complex institutional and regulatory pathways, and challenges with integrating tissue-engineered products and/or stem cell-based therapies into routine clinical practice. Regenerative therapy research requires careful planning, costly resources, special facilities, and dedicated personnel [31,32]. Applying this concept to individual, precision-based regenerative therapies understandably poses a more significant challenge. Current regenerative approaches in breast reconstruction, such as autologous fat grafting and acellular adipose matrices, have demonstrated some clinical efficacy but lack consistent integration with genomic or molecular profiling to individualize treatment, thus limiting their role within the broader framework of precision medicine.

### 4.3. Barriers to Clinical Applications

Key barriers to the clinical application of precision medicine include high costs, limited insurance coverage of testing, and a lack of evidence-based outcomes on the impact of genomic data on surgical outcomes. Broad-based genomic testing (i.e., BRCA testing, Oncotype) frequently encountered in the medical setting is generally covered by insurance, while custom-based genomic tests encountered in this review are likely not to be covered by insurance [33]. To facilitate broader clinical adoption, efforts should focus on the conduction and publication of high-impact clinical trials highlighting the utility of genomic-based tools in enhancing outcomes in breast reconstruction. Thrombophilia testing highlights the importance of genomics in reconstructive decision-making [34]. While universal screening is not routine, identification of genetic-risk patients may allow for tailoring of reconstructive surgical strategies, shorter operations, and patient-specific risk-reduction modalities. For example, if routine thrombophilia testing enhances reconstructive decision-making while also decreasing peri-operative thromboembolic events, this would incentivize insurance coverage of such testing. Positive outcomes from these types of studies can support reimbursement policies and encourage insurance providers to offer coverage for these genomic tests. Standardization of genomic tests from low-power studies to be applied to large populations must also occur.

In addition to navigating financial and logistical barriers, a shift in the current clinical paradigm may be necessary to facilitate changes in the clinical setting. Most surgeons are not trained to interpret molecular, genomic, or biomarker-related data, and enhancing surgeon education on this topic will help mitigate practice pattern behaviors. As evidenced by this review, advances in genomics are most accessible and are at the forefront of precision medicine in breast reconstruction; efforts should focus on the thorough development of clinical application strategies of genomics in precision medicine to allow for further planning and development of targeted and regenerative therapies.

### 4.4. Limitations

There are several limitations of this study to consider. Primarily, this review is scoping in nature and lacks the rigor of traditional systematic reviews. A scoping review format was chosen, given the limited number of published studies on precision medicine in breast reconstruction. This study also lacks the statistical power of a meta-analysis. Critical appraisal of individual sources of evidence was not performed. Quality assessment of included studies was not performed. Potential biases in study selection and classification may have influenced the findings, particularly given the evolving definitions of precision medicine and the subjective nature of study classification. Subjectivity was addressed through inter-reviewer assessment and consultation with senior authors when necessary. Furthermore, limitations in available databases may have resulted in the omission of potentially relevant studies.

### 4.5. Future Directions

This scoping review highlights multiple potential future directions in the research and clinical practice of precision medicine in breast reconstruction. Utilizing genomics and related disciplines, efforts should be dedicated towards the development of real-time screening tests of known biomarkers, as well as the identification of other clinically relevant biomarkers and patient genes associated with adverse outcomes. Gene therapies and potentially therapeutic deliverance of medications are the cornerstone of targeted therapies and should be utilized in breast reconstruction. Individualized stem cell harvesting and customization of growth factors are significant alleyways for progress in regenerative therapies and precision medicine in breast reconstruction.

## 5. Conclusions

In conclusion, this scoping review suggests there is a limited but emerging body of literature on the topic of precision medicine in breast reconstruction with significant promise for clinical translation. Key concepts in precision medicine in breast reconstruction focus on genomics, targeted therapies, and regenerative medicine. Genomic data are the driving force of precision medicine in breast reconstruction, and multiple potential avenues exist to achieve translational applications in the short-term period. Future efforts should focus on translating known genomic data into real-time clinical applications and investing in precision-based research for targeted therapies and regenerative medicine in breast reconstruction.

## Figures and Tables

**Table 1:** Characteristics of Studies on Precision Medicine in Breast Reconstruction.

Citation	Study Design	Population	Intervention/ Outcomes Measured	Key Findings	PM Relevance	PM Category/ Classification
Hickey et al. 2011 [11]	Retrospective clinical study	42 women who underwent post-oncologic BR	• Assessment of persistent postsurgical pain and functional impact • Single nucleotide polymorphism, pain scales, medication use	Trend seen between persistent postsurgical pain and COMT genetic variations	Preoperative genetic screening to tailor pain management strategies for individual patients	• Genomics • Foundational
Nguyen et al. 2013 [12]	In vivo experimental	Rat model of flap ischemia using superficial inferior epigastric artery flap	• Controlled induction of venous and arterial flap ischemia • Biomarkers measured through microarray, qRT-PCR, histology, transcriptomics	Identified five potential biomarkers (Prol1, Muc1, Fcnb, Il1b, Vcsa1) of flap ischemia	Potential for real-time patient-specific monitoring based on personalized biomarkers	• Genomics • Foundational
Sandberget al 2017 [13]	Retrospective cohort clinical study	1931 reconstructed breasts from BC patients who underwent mastectomy between 1999 and 2012	BC subtyping: • Luminal A, B, HER2, HER2-enriched, triple-negative By BC subtype: • Need for radiation • Recurrence-free survival • BR plan	BC molecular subtype can inform optimal timing and type of BR	BC subtype can influence timing (i.e., immediate vs. delayed) of BR	• Genomics • Clinical practice
Basta et al. 2019 [14]	Prospective clinical study	15 breasts with suspected periprosthetic infection	• Alpha Defensin-1 (AD-1) biomarker assay, culture/gram stain, other inflammatory markers • Sensitivity and specificity of AD-1 versus bacterial culture	AD-1 more sensitive and specific for detecting these infections than culture, but difference not statistically significant	Unique biomarkers can be used to detect periprosthetic implant infections in BR and guide management	• Genomics • Foundational
Davis et al. 2020 [15]	Ex vivo experimental study	Rats with BC cells to create locoregional recurrence model	• IFNγ gene transduction via viral vector into autologous tissue vs. sham reconstruction • Tumor burden, survival, molecular mechanism of action, immune response	Reconstructions with IFNγ gene vector had significantly decreased tumor burden and increased survival compared to sham group	Autologous tissue used in BR can be augmented with targeted immunotherapy to reduce BC recurrence in candidate patients	• Targeted therapy • Foundational
Frisell et al. 2023 [16]	Ex vivo clinical study with genetic analysis	BC patients who underwent implant-based BR; 13 irradiated and 12 non-radiated capsules	• Tissue from radiated and non-radiated breast implant capsules analyzed • Genetic expression and immunohistochemistry	200+ inflammatory genes identified, radiated breasts had increased B-cell inflammation and infiltration of breast capsules	Identification of patient-specific genes to guide development of tailored strategies to prevent and/or treat capsular contracture	• Genomics • Foundational
Mao et al. 2024 [17]	Retrospective clinical study	12 patients/15 breasts with capsular contracture	• Analysis of samples of breast capsules • Analysis of genes associated with capsular contracture	PRKAR2B is a novel diagnostic biomarker for breast capsular contracture	Patient biomarkers that may help predict, diagnose, and potentially prevent capsular contracture in BR	• Genomics • Foundational
Anker et al. 2024 [18]	Prospective randomized clinical trial	40 women undergoing post-oncologic autologous BR	• Liberal vs. restrictive post-operative fluid administration • Measurement of urinary biomarkers of kidney stress, TIMP-2 and IGFBP-7	Restrictive fluid administration group had higher TIMP-2 and IGFBP-7 levels indicating increased kidney stress	Tailored fluid management strategies to mitigate risk of kidney injury in BR based on biomarkers.	• Genomics • Foundational
Miller et al. 2025 [19]	Ex vivo clinical study with genetic analysis	7 women with unilateral BC undergoing bilateral tissue expansion and mixed BR (implant on non-radiated side, autologous flap on radiated side) after radiation	• No specific intervention; evaluating radiation-induced fibrosis • Post-radiation breast tissue samples undergo immunohistochemical analysis and RNA sequencing	Multiple genes in breast capsule suggest less microvascularization, increase in immune-related genes and decrease in keratin-related genes in skin	Identification of patient-specific genes to guide development of tailored strategies to prevent and/or treat radiation-induced fibrosis	• Genomics • Foundational

## Data Availability

No new data were created or analyzed in this study.

## Abbreviations

The following abbreviations are used in this manuscript:

BC	Breast Cancer
BR	Breast Reconstruction
PM	Precision Medicine
